# Cardiac organoids: a new tool for disease modeling and drug screening applications

**DOI:** 10.3389/fcvm.2025.1537730

**Published:** 2025-05-20

**Authors:** Ahmed Yaqinuddin, Abdullah Jabri, Abdulaziz Mhannayeh, Bader Taftafa, Mohamed Alsharif, Tasnim Abbad, Jibran Khan, Abdulrahman Elsalti, Raja Chinnappan, Eman A. Alshehri, Alaa Alzhrani, Dalia A. Obeid, Iriya Fujitsuka, Mahmood Khan, Mati ur Rehman, Tanveer Ahmad Mir

**Affiliations:** ^1^ College of Medicine, Alfaisal University, Riyadh, Saudi Arabia; ^2^ International School of Medicine, Istanbul Medipol University, Istanbul, Türkiye; ^3^ Laboratory of Tissue/Organ Bioengineering & BioMEMS, Organ Transplant Centre of Excellence (TR&I-Dpt), King Faisal Specialist Hospital & Research Centre, Riyadh, Saudi Arabia; ^4^ Department of Medical Laboratory Technology, Faculty of Applied Medical Sciences, King Abdulaziz University, Jeddah, Saudi Arabia; ^5^ Division of Basic and Translational Research, Department of Emergency Medicine, The Ohio State University, Columbus, OH, United States; ^6^ Laboratory of Molecular Immunology, Institute for Quantitative Biosciences, The University of Tokyo, Bunkyo-ku, Tokyo, Japan; ^7^ Department of Biological and Biomedical Sciences, Aga Khan University, Karachi, Pakistan

**Keywords:** cardiac organoids, 3D culture, decellularized matrix, disease model, drug screening

## Abstract

Cardiac organoid is a miniature and simplified three-dimensional (3D) cellular model system grown from progenitor cells or stem cells that more accurately mimic the significant biological characteristics and functions of the normal cardiac system than conventional two-dimensional (2D) models. With continued advances in 3D culture approaches, the cardiac organoid models produced through self-organization strategy following developmental induction conditions exhibit higher metabolic similarities and physiological relevance. Increasing evidence demonstrates that cardiac organoids based on the *in vitro* model system are useful platforms for studying human cardiac biology and pathophysiology. Despite significant advancements, the development of cardiac organoids has not progressed as far as other types of organoids due to the intricate cellular structure and microenvironment of the heart. In this review, we highlight the current classification and bioengineering strategies for establishing cardiac organoids using Matrigel and decellularized extracellular matrix derived culture platforms followed by a review of contemporary reports of their use in development biology, disease modeling, drug testing and efficacy evaluation. We also shed the light in the current limitations and future perspective of the cardiac organoid to motivate future research and accelerate the widespread adoption of organoids platforms.

## 1 Introduction

Cardiovascular diseases (CVD) are the leading cause of mortality and substantially contribute to healthcare and economic burdens worldwide (1). Despite enormous funding investments and implementation of various drug development strategies, ninety percent of new drugs fails in clinical trials (2). Cardiovascular disease and cancer have the lowest success rates in discovering new drug candidates, largely due to adverse effects of the therapeutic agents that result in clinical and subclinical cardiotoxicity (3–6). Because the elucidation of disease pathogenesis and high-throughput screening are of paramount importance in the development of new drugs, various 2D and 3D cell model systems have been published over the past several decades (7–9). In the traditional setting, cells are routinely cultured in 2D format for the purposes of disease research and drug screening, but 2D models inherently lack the structural complexity of their *in vivo* counterparts (10–12).Therefore, intensive research efforts have been made to develop next-generation culture methods that mimic important features of native tissues, such as the cellular and acellular microenvironment and cell-cell interactions when performing 3D cell culture experiments *in vitro*. The rapidly evolving fields of organoid engineering and biological model systems are continually providing new insights into basic experimental biology, human disease mechanisms and drug response efficacy research and are driving new therapeutic innovations (13, 14). Cardiac organoids are organized structures that self-assemble into miniaturized models composed of progenitor cells, cardiomyocytes, endothelial cells, and fibroblasts in a three-dimensional microenvironment that mimic the *in vivo* native organs (15, 16). Unlike traditional 2D myocardial culture systems, cardiac organoids recapitulate the human-specific aspects of heart histogenesis, physiology and developmental trajectory. Therefore, cardiac organoids have grown in popularity because they provide a unique opportunity to model cardiac diseases drug screening, and toxicity testing (17, 18). They are considered as powerful gateway to understand cardiac functions not only from a basic science perspective but also for the development of personalized therapies (19, 20). Towards that end, a wide range of cardiac organoid models have been reported over the past few years, applying a variety of experimental strategies (21, 22). Nonetheless, progress in the study of cardiac organoids is much slower than that of its other counterparts including brain, liver, kidney, and intestinal models (20–25). This represents an opportunity for active research related to the biofabrication techniques for the engineering of different models of cardioids for translational regenerative medicine applications (23–28).

In this glance article, we begin with history of the development of organoid technology and the use of organ-specific biomaterials derived from decellularized hearts in culture for cardiac organoid research. We then summarized their applications as *in vitro* model systems and drug screening tools. Despite the tremendous potentials of organoids models, it is essential to understand the existing hurdles and limitations of organoid technology. Therefore, we highlighted the current challenges and respective advantages of cardiac organoid research for basic and translational applications.

## 2 Timeline and developmental history of organoid technology

The initial efforts to generate organs *in vitro* began with pioneering dissociation-reaggregation experiments. In these early studies, Henry Van Peters Wilson showed that sponge cells, when mechanically separated, have the ability to come together again and self-organize, ultimately forming a complete organism (29). Several decades after this initial experiment, multiple research teams conducted dissociation-reaggregation studies, successfully creating various types of organs from separated cells of the amphibian pronephros and chicken embryos (30, 31). However, the initial observation of *in vitro* tissue-like colonies formation occurred through the co-culture of keratinocytes and 3T3 fibroblasts (32). An important milestone in this journey was the development of the differential adhesion hypothesis, which was prompted by the observation that cells, upon mechanical dissociation and subsequent aggregation, could autonomously reorganize into the original tissue structures from which they were derived. This phenomenon underscored the inherent capacity of cells for spatial organization and tissue reconstruction. Advancements in stem cell biology further elucidated the potential of stem cells to differentiate and organize into organ-like structures *in vitro*, as evidenced by the formation of teratomas and embryoid bodies. These entities demonstrated the ability of differentiated cells to form arrangements mimicking various tissue types. From the end of the 19th century (1998) to the beginning of the 20th century (2006), intensive research on stem cell technology, especially human embryonic stem cells (hESCs) and induced pluripotent stem cells (iPSCs), triggered a wave of studies on the mechanisms and fate of stem cells in different culture environments (33, 34). In 2009, Hans Clever and team found that individual intestinal stem cells expressing leucine-rich repeat-containing G-protein coupled receptor 5 (Lgr5) stem cells produce crypt-villus structures *in vitro*, independent of mesenchymal niche (i.e., stroma), marking the creation of the first organoids. Their findings showed that LGR5+ stem cells are involved in rapid regeneration of intestinal tissue and can self-organize the intestinal crypt-villus units from a single stem cell without requiring a surrounding cell niche (35). Since then, a variety of organoid models have been reported by different research groups, including intestinal, brain, heart, kidney, and liver, among others (35–39). These studies laid the groundwork for numerous other studies on organoids across various systems, such as the mesendoderm (including organs like the stomach, liver, pancreas, lung, and kidney) and neuroectoderm (such as the brain and retina), employing either adult stem cells (ASCs) or pluripotent stem cells (40).

## 3 Procedural requirements for generating of cardiac organoids

**Figure 1:**
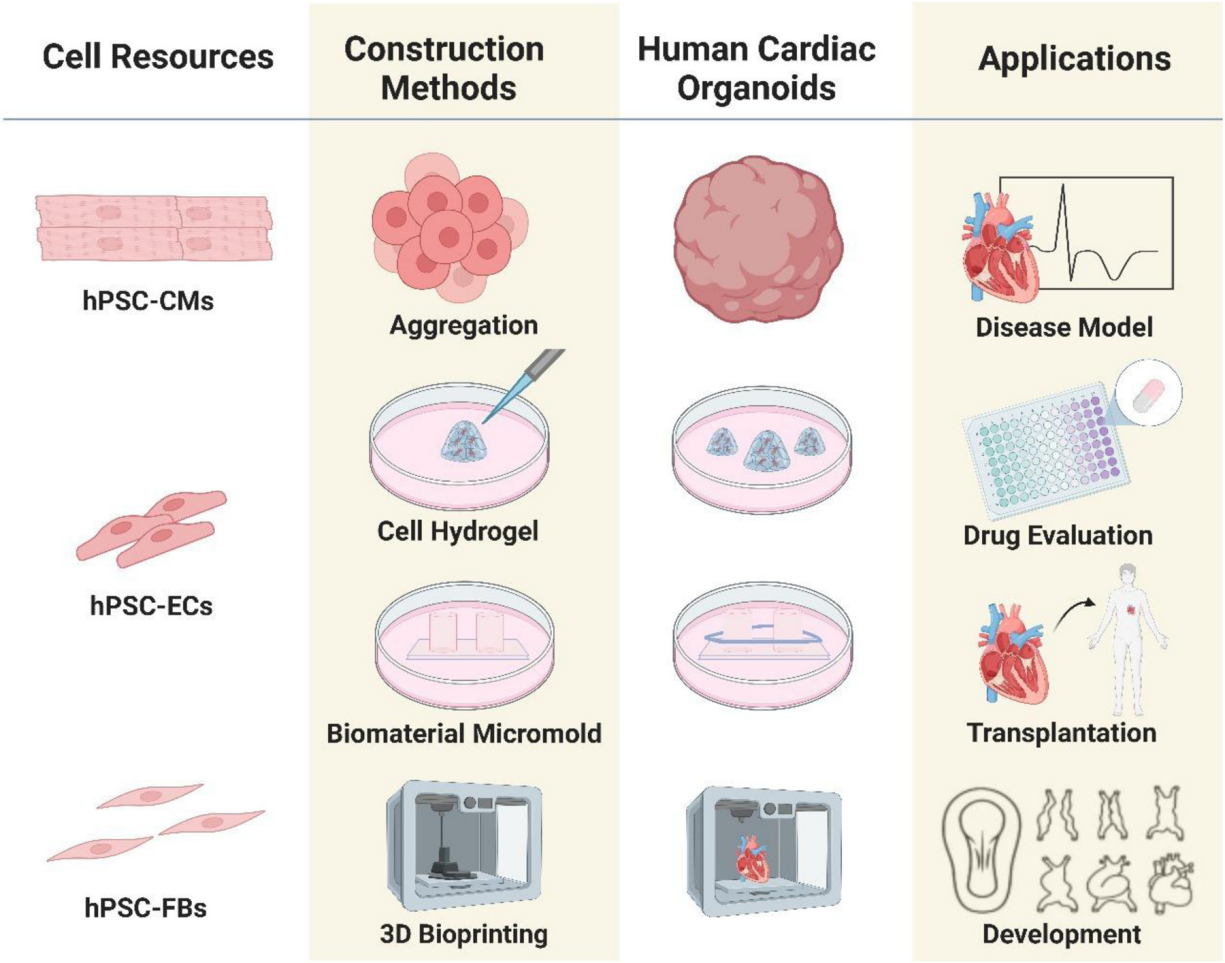
Outline of the process of organoids culture techniques and bioengineering strategies: organoids can be generated from different cell sources isolated from human/animal tissue samples. The construction methods play crucial role in the proper growth and development of organoid models.

The primary basis for the development of organoid culture relies on the ability of homogeneous cells to self-organize and mimic the key features of the source tissue. The key steps in self-organization rely heavily on a series of highly regulated signaling pathways that play an important role in the self-organization and cell differentiation processes in the developmental cascade of pattern formation through a variety of morphogenetic rearrangements. The traditional method of organoid culture, expansion and development involves the enzymatic digestion of tissue fragments and derivation of stem cells, progenitor cells, followed by culturing them in a 3D microenvironment using growth factor-conditioned medium (15). A variety of morphogens are used to culture of cardiac organoids, including Wnt-3a epidermal growth factor (EGF), fibroblast growth factor (FGF), R-spondin, gastrin, noggin, Human Bone Morphogenetic Protein 4, Human Activin-A, Insulin, B-27 supplement, L-ascorbic acid 2 phosphate, Aprotinin, Palmitic acid. The methods for creating *in vitro* cardiac organoids using stem cells, progenitor cells, cardiomyocytes (CMs), endothelial cells (ECs), and cardiac fibroblasts, fall into two categories: scaffold-based and scaffold-free techniques (Figure 1). Scaffold-based methods utilize biomaterials like hydrogels or decellularized bioscaffolds, whereas scaffold-free techniques typically involve promoting the spherical aggregation of cultured cells in an anti-adhesive setting, in addition to some newly emerged techniques, used to facilitate cardiac organoid formation, including microarray technology, 3D bioprinted models, and scaffolds based on electrospun fiber mats. Scaffold-free 3D cultures are created by the self-assembly of cells, while scaffold-based cultures use hydrogels or other scaffolds to support tissue replicas. In either case, the cells are organized into a three-dimensional structure, which is essential for preserving their morphology, phenotype, and polarity. Scaffold-free systems can create artificial 3D heart tissues that maintain mechanical integrity without the need for external support (41). This paper will focus solely on the development of cardiac models using scaffold formation methods with decellularized materials. To understand the basic concepts and operating principles of biomaterials and bioengineering strategies suitable for organoids applications, the readers can refer to more specialized reviews (42, 43).

## 4 Necessity to develop Matrigel alternative biomaterials for organoids research

A large portion of organoids have been grown in Matrigel, a complex and poorly defined biomaterial produced from the secretions of Engelbreth-Holm-Swarm mouse sarcoma cells (44). Although affordable and versatile, Matrigel is highly complex; a proteomic study reveals that it comprises more than 1,800 distinct proteins (45). It is challenging to determine the signals required for organoid construction and function due to Matrigel's vague nature, which is made more challenging by lot-to-lot variances in Matrigel (46, 47). Additionally, Matrigel based culture environment may lack some of the constituents required for the production of healthy organoid models. For example, gut organoids generated and maintained in Matrigel lack the distinctive villous structure of mammalian intestines, which may be caused by insufficient laminin-511 and associated components (48, 49). Also, it is becoming increasingly evident that the permeabilities and mechanical characteristics of 3D environments can significantly impact the development of cells (50), organoids (51), tissues, and organs (52, 53). The mechanical behaviors-like pore size, elasticity, stress relaxation moduli, and creep compliances (54, 55) -cannot be readily distinguished from the biochemical signal outputs in the Matrigel-based culture environments. Moreover, Matrigel samples have diverse mechanical characteristics; certain parts of these hydrogels are been known to exhibit elastic moduli and stiffness related properties that are considerably higher than the average elastic modulus of the sample (56, 57). Finally, due to possible immunogenicity, Matrigel's origins in mouse cells make it unsuitable for use in clinical transplantation of humans (58). With all these shortcomings, there is growing demand to create Matrigel-free culture techniques for the growth and maintenance of organoids. Extracellular matrix (ECM) proteins function as an adhesive substrate, a signaling cue source, and a growth factor sequestration mechanism during organ development (59).

### 4.1 Decellularized extracellular matrix as suitable material for cardiac organoids

Because of its resemblance to the original tissue, cardiac decellularized extracellular matrix (dECM) has been studied as a potential strategy in tissue-regenerative medicine (60). In a dECM, the basic characteristics of the original tissue can be preserved (60–63). Therefore, cardiac dECM offers myocardiocytes the ideal biomimetic environment in which they can proliferate and repair damaged cardiac tissue (64). There are two types of decellularization methods: chemical and mechanical. Hydrostatic pressure or freeze-thawing are examples of mechanical techniques used to eliminate genetic materials and cellular components. Due to the partial elimination of genetic elements, these approaches are considered advantageous in terms of preserving, biomechanical and biochemical features, but may provoke immunogenicity (65). On the other hand, chemical methods lyse cells by displacing the phospholipid cell membranes with surfactants, acids, and bases. These chemicals eliminate undesirable molecules entirely but can also harm structural and signaling proteins necessary for cell regulation (66). In order to maintain the essential features of dECM, a combination of chemical and mechanical techniques can be used, taking into account the benefits and drawbacks of each method, as indicated earlier (67). The ability of various types of stem cells, including induced pluripotent stem cells (iPSCs), mesenchymal stem cells (MSCs), and embryonic stem cells (ES cells), to promote spontaneous tissue repair after seeding with dECM indicates that acellular ECM-based materials may be useful in cardiac organoids research (67–70).

Certain organoids have been cultured on dECM derived from human or animal donors, with the aim of precisely replicating the composition, structure, and vascularization of native ECM throughout organ development. The target tissue determines the decellularization techniques used, which makes them difficult to generalize (71). Even though xenogeneic ECM may trigger immunological reactions, this danger can be significantly decreased with the right preparation methods (72). FDA-approved similar animal-derived ECM scaffolds are used in clinical applications, including orthopedic implants, face reconstruction, and cardiac valve replacement (59, 73). Decellularized extracellular matrix can also offer extra signals that promote the repair of injured tissue, ultimately stabilizing the organoid transplant and enhancing its functionality (74).

### 4.2 Pros and cons of decellularized extracellular matrix

ECM-based techniques that have been decellularized can rapidly replicate organ function. There is no need for the surface chemistry modification of the ECM because many, if not all, of the chemical signals necessary for the engineering of a spatially defined organ (including glycoproteins that are difficult to introduce) are already present. A decellularized extracellular matrix preserves compositional variations between the basal and apical areas (62). Decellularized ECMs also have some drawbacks. Most notably, the availability of human or animal donors limits the amount of ECM that can be studied, and donor quality can impact the quality of ECM. For instance, the architecture of fibrotic or emphysematous lung tissue has changed and become harder. These modifications may result in cells that do not survive in culture for more than one week (75, 76) or in significant alterations to the phenotype of seeded cells that can survive (76). On the other hand, myocardial infarction is known to induce changes in the biophysical and biochemical properties of the ECM; changes in surface chemistry make the ECM stiffer and more susceptible to topological changes. Nevertheless, when cells are grown on infarcted tissue, they secrete an abundant amount of immunomodulatory as well as pro-survival growth factors (61). Although myocardial infarction appears to increase the survival of incorporated cells, it is essential to consider the detrimental effects of other unhealthy tissues on the formation and maintenance of organoid models (62). Furthermore, batch-to-batch variability remains a challenging problem, even for tissue derived from healthy donors. The physicochemical and mechanical properties of decellularized ECM are difficult to control and modify due to its dynamic and complex network, limiting the efficiency of the matrix and its wider adoption. In addition to being chemically ambiguous, decellularized extracellular matrices often lack established drivers of differentiation. Aggressive decellularization may eliminate surface proteoglycans required for effective organoid formation (77). Another challenge is that different decellularization techniques have different levels of success in eliminating cells or other immunogenic species, which can lead to different host immunological reactions and implant failure in clinical trials (78). Lastly, there are times when PSC differentiation into organ-specific progenitor cells that are subsequently incorporated into the decellularized matrix needs a further step.

## 5 Applications of cardiac organoids

Organoid technology has recently brought a dramatic shift to biomedical research by creating 3D models that replicate cellular heterogeneity, structure, and functions of tissues (40). In recent years, organoids have been used widely in disease modeling and drug discovery in several organs, such as the brain (79), the kidney (39), the liver (80, 81), and the intestine (82). However, due to the heart's complex structure and vascularization, the progress of developing cardiac organoids has been slower than that of other organs. Regardless of these limitations, human cardiac organoids are widely considered as a novel model system for studying cardiac diseases (Table 1), drug screening and cardiotoxicity testing (84–91) (Tables 1, 2).

**Table 1:** Cardiac organoids for disease modeling.

Cardiac organoids for disease modelling						
Model type	Media cocktail	Cell source	Engineering method	Disease category	Outcome	Ref.
Cardiomyopathy	Standard cardiomyocyte medium with bFGF, VEGF	iPSCs, hiPSCs	3D bioprinting with scaffold	HCM, DCM, ACM	Enhanced cell viability and function	(83, 84)
Myocardial ischemia	Ischemia-specific medium with protective factors	iPSCs	Hydrogel embedding method	AMI	Improved nutrient diffusion	(85, 86)
Cardiovascular injury	Injury model medium with added cytokines	hiPSCs, Human embryonic stem cells	Self-assembly in microfluidic devices	Acute freezing injury, COVID-19 impact	Effective organoid formation	(87)
Congenital cardiac disease	Genetic disease medium with tailored growth factors	iPSCs	Bioreactor for dynamic culturing	Genetic induced	Higher replication of *in vivo* conditions	(88)
Arrhythmia	Arrhythmia-specific medium with electrophysiological supplements	hiPSCs	Patch clamp technique for arrhythmia	Inherited arrhythmias	Accurate simulation of arrhythmic conditions	(89, 90)

The table above highlights the diversity in the methodologies and applications of cardiac organoids in disease modeling.

**Table 2:** Cardiac organoids for drug screening.

Cardiac organoids for drug screening applications							
Model type	Media cocktail	Cell source	Engineering method	Drug screening	Toxicity testing	Outcome	Ref.
Cardiomyopathy	Standard cardiomyocyte medium with bFGF, VEGF	iPSCs, hiPSCs	3D bioprinting with scaffold	Drug response analysis	Cardiotoxicity studies	Enhanced cell viability and function	(83, 84)
Myocardial ischemia	Ischemia-specific medium with protective factors	iPSCs	Hydrogel embedding method	Ischemia drug effects	Ischemic toxicity	Improved nutrient diffusion	(85, 86)
Cardiovascular injury	Injury model medium with added cytokines	hiPSCs, Human embryonic stem cells	Self-assembly in microfluidic devices	Injury impact of drugs	Toxic impact assessment	Effective organoid formation	(87)
Congenital cardiac disease	Genetic disease medium with tailored growth factors	iPSCs	Bioreactor for dynamic culturing	Genetic disorder treatments	Toxicity in genetic therapies	Higher replication of *in vivo* conditions	(88)
Arrhythmia	Arrhythmia-specific medium with electrophysiological supplements	hiPSCs	Patch clamp technique for arrhythmia	Arrhythmia drug response	Arrhythmogenic toxicity	Accurate simulation of arrhythmic conditions	(89, 90)

The table above highlights the applications of cardiac organoids for drug screening and toxicity testing.

## 6 Future directions: enhancing maturation and improving fidelity to native tissue

Advancements in cardiac organoids that allow appropriate structural and functional elements of the normal heart are imperative. These include the formation of chambers, myocardium thickness, and contractibility. One recent advancement in cardiac chamber formation using organoids includes the hHO model by Lewis-Israeli et al., which involves self-organization into multiple miniature chambers via BMP4 and Activin A (37). Additionally, Lin et al. successfully established a “human-heart-in-a-jar”, demonstrating an electrically and mechanically functional miniature ventricle (83). Although these characteristics provide an obvious advantage for studying diseases and drug interactions over using simpler 2D or 3D models, they lack essential features including left-right symmetry, conductance, and valves (91). Bioreactors have been suggested in the literature to improve the maturity of cardiac organoids (92). They work by improving media circulation, which leads to elevated uptake of nutrients, and providing mechanical stretch stimuli, which promote organoid maturation (93, 94).

Lastly, poor vascularization of cardiac organoids has been observed in several models, with some even developing necrosis from limited perfusion (24). Organ-chip systems have addressed this drawback, allowing 3D microchannel scaffolds supporting millimeter-thick cardiac tissue that is optimally perfused (24, 95). A limitation of these systems is the inability to replicate the 3D configuration and multicellular composition of normal cardiac tissue. Indeed, a multicellular composition, incorporating endothelial cells and fibroblasts, enables enhanced disease modeling and cardiomyocyte maturation (87). A potential solution to this has been observed with a Heart-on-Chip (HoC) model described by Cofiño-Fabres et al., involving the addition of hiPSC-derived cardiomyocytes with other cells typically found in cardiac tissue (96). Another alternative to improve vascularization involves the development of hiPSC-derived self-assembling vascular spheres, followed by encasing with hiPSC-derived cardiomyocytes (97).

## 7 Conclusion

In summary, while cardiac organoids represent a significant advancement in heart disease modeling and therapeutic research, challenges remain in fully replicating the complexity of the heart's structure and function. The absence of a cardiac conduction system, functional vascularization, and the lack of standardized fabrication methods are key obstacles that hinder the development of more accurate and reliable models. However, with ongoing advancements in 3D modeling techniques, such as Heart-on-Chip systems, these limitations are gradually being addressed. As the field progresses, cardiac organoids hold great promise for improving disease modeling, drug screening, and personalized medicine, paving the way for more effective treatments and better understanding of cardiovascular diseases. Continued research into fabrication methods, tissue engineering, and functional integration will be essential for realizing the full potential of cardiac organoids in both clinical and preclinical applications.
